# Editorial: Brain-liver axis and glutamate homeostasis, volume II

**DOI:** 10.3389/fendo.2026.1812353

**Published:** 2026-03-10

**Authors:** Ana Catya Jimenez-Torres, Mustapha Najimi, Arturo Ortega

**Affiliations:** 1 Department of Pharmacology and Toxicology, Medical College of Georgia, Augusta University, Augusta, GA, United States; 2 Laboratory of Pediatric Hepatology and Cell Therapy, Institute of Experimental and Clinical Research (IREC), UCLouvain, Brussels, Belgium; 3 Departamento de Toxicología, Centro de Investigación y de Estudios Avanzados del Instituto Politécnico Nacional (Cinvestav-IPN), Ciudad de México, Mexico

**Keywords:** acute-on chronic liver failure, brain-liver axis, cognitive disorder, encephalopathy, glutamate

## Introduction

Accumulated evidence from animal models supports the crosstalk between the brain and liver in the regulation and sensing of glucose, lipids, and hormones (1). Both organs respond dynamically to environmental stimuli, sustaining not only metabolic balance- relevant for food intake and energy storage (2)- but also cognitive functions and emotional stability (3, 4). Anatomically, the *so-called* brain-liver axis is a bidirectional communication pathway that occurs through parasympathetic and sympathetic innervation between both organs (5–7). It is well known that the liver is essential for maintaining metabolic homeostasis; its failure might impact brain function *via* endocrine, neural and metabolic signals (4). Meanwhile, efferent projections from the hypothalamus and *medulla oblongata* to the hepatic branch *via* the vagus and splanchnic nerves control the secretion of hormones and neuropeptides that are key for liver regeneration (6–9).

This Research Topic provides an overview of the metabolic crosstalk between the liver and the brain, focusing on bile acids as key signaling molecules in the regulation of mood-state in individuals. Furthermore, an update on the signature transcriptome of specific cell types across the brain-liver axis is also reported. The plausible molecular mechanisms *via* non-coding RNAs that impact differential gene expression patterns in the etiology of neuronal and liver injuries highlight common targets within the brain-liver axis. Finally, clinical evidence consolidates the importance of addressing the factors involved in the pathophysiology of brain disorders during liver disease.

## Metabolic signaling between the liver-brain axis: bile acids as mediators of anxiety disorders

The vagus nerve is a key component in liver-brain communication and plays a role in the development of cognitive disorders during liver failure. Chen et al. review the contribution of bile acids to the development of anxiety disorders. This review emphasizes that bile acids can induce anxiety-like behavior mainly through two mechanisms: first, by directly binding to their receptors expressed across the blood-brain barrier; and second, by forming receptor-hormone complexes that enter systemic circulation *via* the enterohepatic circulation and reach specific receptors in the brain. Therefore, disruption of the synthesis, metabolism and circulation of bile acids might be critical factors in the occurrence of anxiety behaviors during liver injury. Clinical studies have shown a positive association between chronic liver diseases and the incidence of anxiety and depression (10). In this context, the authors describe specific ligands such as FXR, TGR5, and S1PR2 which can be activated by various bile acids, and are expressed in both liver tissue and the brain. These proteins can stimulate the transcriptional machinery that leads to the expression of growth factors and the secretion of small peptides that, after systemic circulation, can reach brain regions such as the cerebral cortex, hypothalamus, and hippocampus. This process regulates not only inflammatory or oxidative stress responses but might also influence mood behaviors.

## Signature transcriptome across the brain-liver axis and non-coding RNAs as novel targets for treating neurological disorders in liver disease

The astrocytes and hepatic stellate cells (HSCs) are specific brain and liver cell types, respectively, closely involved in the early response to injury in both organs (11). Jimenez - Torres et al. report a comprehensive update on the transcriptome of activated HSCs during liver injury and contrast it with that of reactive astrocytes in neuronal diseases. An overview of the pseudotime trajectory of the genes expressed in HSCs- from quiescent state to myofibroblast-like phenotype during various liver diseases such as non-alcoholic steatohepatitis, fibrosis, and hepatocellular carcinoma- and the atlas of genes related to reactive gliosis in neurocognitive diseases such as Alzheimer’s disease, Parkinson’s disease, and hepatic encephalopathy suggest that a plausible combination therapy targeting GFAP, miR-455-3p, miR-140, miR-148a-3p, and the long non-coding RNA HOTTIP- key molecules across the brain- liver axis- might be a promising therapeutic strategy for tissue repair in neuronal and hepatic diseases.

## Clinical implications

The development of brain disorders during acute liver injury has been well described (12–16). However, although it is relatively uncommon, neurological deterioration can also be observed in patients with acute-on chronic liver disease. In a case report, Tang et al. show that, similar to the signs during acute liver injury, the levels of ammonia, serum lactate, and procalcitonin increase 11-, 5-, and 8-fold, respectively, in a patient with cirrhosis-related acute-on chronic liver failure compared to the reference values. The patient presented with liver and kidney dysfunction that was accompanied by loss of grey-white matter differentiation. In this case report, despite the reduction of serum ammonia levels following treatment, cerebral and cerebellar edema with bilateral uncal herniation was observed 72 hours post-admission, leading to brain death. This suggests two important possibilities: i) ammonia is not the only factor involved in the accumulation of fluid in the brain and intracranial hypertension; ii) despite the treatment, the cytotoxic effects in the brain during the initial phase of hepatic encephalopathy in acute-on chronic liver disease may be irreversible, resulting in a poor prognosis. For example, hyperammonemia indirectly drives various mechanisms of neurotoxicity, such as increased glutamate levels in the synaptic cleft, cellular swelling due to loss of osmotic balance, inflammation, and oxidative stress (17). The expression profile of small noncoding RNAs (miRNAs) in the cerebral cortex of mice subjected to drug-induced acute liver injury has shown alterations in miRNAs specifically involved in glutamatergic synapses, Wnt, MAPK and PI3K-Akt signaling pathways following acute liver failure-induced hepatic encephalopathy (18). Moreover, modifications in the protein expression of glutamate transporters and neuronal loss in the cerebellum have been reported in the early stages of drug-induced acute liver injury in mice (19).

Hyperammonemia is a common feature of liver disease. However, rare cases can present high levels of ammonia leading to neuronal dysfunction and coma in patients with normal liver function (20). Hashimoto’s encephalopathy is an uncommon neurological disease associated with autoimmune thyroiditis, often occurring in patients with normal or mild clinical or subclinical hypothyroidism. Its diagnosis is challenging due to nonspecific symptoms in the absence of any infection or tumoral causes. The pathogenesis is thought to involve autoimmune mechanisms affecting the brain. In this Research Topic, Croce et al. elaborate on these aspects, emphasizing the importance of recognizing this condition for timely treatment.

## Conclusion

Taken together, the reports in this *e-book* collection highlight the importance of understanding the intricate mechanisms underlying complex crosstalk in the brain-liver axis which is essential for developing innovative therapies aimed at enhancing neurological outcomes and prognosis in patients with hepatic failure.
